# Detection of dating violence among university students in health sciences: a multicentric cross-sectional study in Spain and Colombia

**DOI:** 10.3389/fgwh.2026.1831359

**Published:** 2026-07-14

**Authors:** Montserrat Puig-Llobet, Maria Aurelia Sánchez-Ortega, Marta Prats-Arimon, Zaida Agüera, Dolors Rodríguez-Martín, Carmen Moreno Arroyo, Elena Maestre, Maria Teresa Lluch-Canut, Juan F. Roldán Merino, Antonio Moreno-Poyato, Maria Angeles Saz Roy, Susana Mantas-Jiménez, Pepita Giménez-Bonafé, Núria Vergés Bosch, Núria Ferran Ferrer, Adrien Faure-Carvallo, Cristina Manzanares Cespedes, Wilson Astudillo-Rozas, Núria Rodríguez Ávila, Cristina Casanovas-Cuellar, Ariadna Huertas-Zurriaga, Elisabet Torrubia-Pérez, Gerard Mora-López, Judit Vives-Espelta, Montserrat Sanromà-Ortiz, Judith Roca, Zuleima Cogollo, Rafael Tuesca-Molina, Sara Sanchez-Balcells

**Affiliations:** 1 Departament de Salut Pública, Salut Mental I Maternoinfantil, Facultat D’Infermeria, Universitat de Barcelona, L’Hospitalet de Llobregat/Barcelona, Espanya; 2 Research Group in Mental Health, Psychosocial and Complex Nursing Care (NURSEARCH), Facultat D’Infermeria, Universitat de Barcelona, L’Hospitalet de Llobregat/Barcelona, Spain; 3 Department of Professional Foundations and Health Management, Faculty of Health Sciences and Well-being, University of Vic, Vic/Barcelona, Spain; 4 Department D'Infermeria Fonamental I Clínica, Facultat D’Infermeria, Universitat de Barcelona, L’Hospitalet de Llobregat/Barcelona, Espanya; 5 Research Group in Gender, Identity and Diversity (GENI), University of Barcelona, Barcelona, Spain; 6 Campus Docent Sant Joan de Deu, Universitat de Vic-Universitat Central de Catalunya (UVIC-UCC), Sant Boi de Llobregat/Barcelona, Spain; 7 Research Group Health and Healthcare, Department of Nursing, University of Girona, Girona, Spain; 8 Department of Physiological Sciences, Faculty of Medicine and Health Sciences, Bellvitge Campus, L’Hospitalet de Llobregat, Barcelona, Spain; 9 Department of Sociology, Faculty of Economics and Business, University of Barcelona, Barcelona, Spain; 10 Faculty of Information and Audiovisual Media, Universitat de Barcelona, Barcelona, Spain; 11 Human Anatomy and Embryology Unit, Experimental Pathology and Therapeutics Departament, Bellvitge Campus, L’Hospitalet de Llobregat, Barcelona, Spain; 12 Nursing Department, Faculty of Medicine, Universitat Autònoma de Barcelona, Bellaterra, Spain; 13 NURECARE Research Group, Germans Trias I Pujol Research Institute, Hospital Germans Trias I Pujol, Institut Català de la Salut, Badalona, Spain; 14 Departament D’Infermeria, Grup de Recerca SGR 161 Infermeria Avançada “Caring”, Facultat D’Infermeria, Universitat Rovira I Virgili, Tarragona, Spain; 15 Departament of Nursing and Physiotherapy, University of Lleida, Campus Igualada, Lleida, Spain; 16 Health Education, Nursing, Sustainability and Innovation Research Group (GREISI), GREISI IRBLL, Lleida, Spain; 17 Research Group “Care for the Health of Communities”, Faculty of Nursing, University of Cartagena, Cartagena, Colombia; 18 Departament of Public Health, Universidad del Norte, Barranquilla, Colombia

**Keywords:** Colombia, cross-sectional studies, health occupations, intimate partner violence, prevalence, Spain, students

## Abstract

**Introduction:**

Dating violence (DV) represents a critical public health challenge that disproportionately affects adolescents and young people. Among Health Sciences students, early detection is doubly important, both for their personal wellbeing and for their future role as frontline professionals in the identification of victims. The aim is to determine the prevalence and dynamics of dating violence (DV) among Health Sciences university students in Spain and Colombia, analyzing patterns of victimization and perpetration.

**Material and methods:**

This multicenter cross-sectional study included 511 Health Sciences students from eight universities in Spain and Colombia. DV was assessed using the Multidimensional Dating Violence Scale (EMVN) developed by García-Carpintero et al. (2018). The study was approved by the Research Ethics Committees of all participating institutions.

**Results:**

The findings reveal that control and surveillance, together with sexual violence, are the predominant dimensions, far exceeding physical violence. Digital control behaviors (“persistently sending messages through social media”) and subtle grooming behaviors (“giving unsolicited gifts or favors”) emerged as the most frequently reported indicators. The bivariate analysis revealed critical gaps: men reported significantly higher levels of perpetration of physical and sexual violence. In addition, the geographical context proved to be a determining factor, showing significant cultural variations in how the phenomenon manifests.

**Conclusions:**

The results highlight that dating violence in this population is predominantly psychological and related to control. It is imperative to integrate education on equality and awareness of “warning signs” into the academic curriculum to transform these students into professionals capable of breaking the cycle of violence.

**Implications:**

This pioneering study provides an international perspective on the vulnerability and perceptions of future health professionals. Its multicenter nature makes it possible to design intervention strategies adapted to specific sociocultural contexts, thereby strengthening the response capacity of health systems to gender-based violence.

## 1 Introduction

Dating violence (DV) is a form of intimate partner violence (IPV) that encompasses the perpetration of emotional, physical, or sexual abuse within the context of a dating relationship (1, 2). Furthermore, DV is commonly conceptualised as a specific manifestation of intimate partner violence occurring in non-cohabiting or early-stage relationships. Within contemporary theoretical frameworks (3), DV is frequently situated within the broader construct of gender-based violence, as it reflects underlying power imbalances and socially constructed gender norms that shape interpersonal relationships. From a feminist and socio-ecological perspective, gender-based violence is understood as a structural phenomenon rooted in historical and systemic inequalities between men and women, which may also manifest in adolescent and young adult relationships.

Although dating violence can occur across different types of relationships and affect individuals of all genders, a substantial body of research highlights that its dynamics, meanings, and consequences are often gendered, with women disproportionately affected by certain forms of victimisation, particularly psychological and sexual violence (3, 4). Therefore, analysing dating violence within a gender-based violence framework allows for a more comprehensive understanding of its sociocultural determinants and its role within broader patterns of inequality and power relations (5).This phenomenon represents an increasingly prevalent social and public health problem among adolescents and young adults (6). The issue of gender-based violence and violence within intimate relationships also constitutes a central concern for feminist scholarship and gender studies, which have emphasized the need to make these forms of violence visible from intersectional and structural perspectives.

This type of violence may manifest through: (a) physical violence, which includes hitting, pushing, kicking, or any other form of bodily aggression; (b) emotional or psychological violence, such as insults, humiliation, manipulation, threats, excessive control, or isolating a partner from their social and family environment; (c) sexual violence, which includes any non-consensual sexual act or pressure to engage in sexual activities; and (d) economic violence, such as controlling a partner’s finances, preventing them from working, or restricting their access to their own financial resources.

Although earlier research has primarily focused on intimate partner violence in adult populations, a substantial body of literature over the past two decades has specifically addressed dating violence among adolescents and young adults, with significant contributions from researchers such as Foshee, Wolfe, and Temple (7–9). With regard to gender differences, the evidence suggests a complex and nuanced pattern. While some studies report similar rates of victimisation across genders for certain forms of dating violence, particularly psychological aggression (3), important differences emerge in terms of severity, impact, and context. Women are more likely to experience more severe forms of violence, including sexual coercion and violence associated with greater physical and psychological consequences (5). Therefore, a gender-informed perspective remains essential to fully understand the nature and implications of dating violence.

The findings of the 2021 systematic review by Tomaszewska and Schuster indicate that psychological violence is the most prevalent form of dating violence in adolescent relationships in Europe, characterized by considerable variability in reported prevalence rates. Regarding perpetration, the studies analyzed report ranges between 10% and 92% among women, whereas among men the levels range from 7% to 87% (10). This wide variability in the data, particularly the upper limit of 92%, has been attributed to the inclusion of low-intensity indicators in certain measurement instruments, such as overt jealousy or insults during episodes of conflict. With respect to physical violence, reported rates range from 4.7% to 32.9% among women and from 7.3% to 29.8% among men. Sexual violence also shows substantial variability, with prevalence estimates ranging from 7.8% to 41% among women and from 6.1% to 31.0% among men (11). Similar patterns are also observed regarding perpetrated violent behaviors. This wide dispersion of findings is consistent with other international studies, in which the overall prevalence of dating violence ranges between 27.7% and 70.7%, although many of these studies do not specify the type of violence assessed nor incorporate a gender perspective (10). In addition to European data, several studies conducted in Colombian populations have documented the prevalence and characteristics of dating violence among adolescents and young people. For example, Rey Anacona (12) reported significant levels of psychological, physical, and sexual violence in adolescent relationships, highlighting the normalization of certain controlling and coercive behaviours. More recent studies, such as Pérez-Ruiz et al. (13), have confirmed the persistence of these patterns among university populations, emphasizing the role of sociocultural factors in shaping the perception and reporting of violence. Furthermore, a large-scale study conducted by Rey et al. (14) with 2,049 adolescents across five Colombian cities identified high prevalence rates of dating violence, particularly in psychological forms, and pointed to challenges in recognising these behaviours as abusive. These findings underscore the importance of considering contextual and cultural differences when analysing dating violence across countries.

In the Spanish context, according to a recent study conducted by the Government Delegation against Gender Violence, one in five young women has experienced some form of gender-based violence in their intimate relationships. These findings highlight the persistence of sexist attitudes among young people, as evidenced by the fact that 25% of young men justify controlling behaviors or jealousy as expressions of affection (11).

At the same time, a significant increase in gender-based violence in the digital sphere has been identified, manifested through monitoring of social media, constant messaging, or demands for personal passwords (11).

This form of violence can have significant implications for the physical and mental health of those involved (15). In terms of victimisation, individuals experiencing dating violence may present bodily injuries such as bruises, fractures, wounds, and other harm resulting from aggression. In addition, various physical health difficulties, including chronic pain and sleep disturbances, have been reported in association with these experiences (16).

In the emotional domain, individuals exposed to dating violence may report symptoms such as anxiety, depressive manifestations, and post-traumatic stress-related difficulties. Lower self-esteem, feelings of guilt or shame, and difficulties in establishing trusting relationships have also been described as difficulties associated with dating violence (17). At the social level, affected individuals may experience challenges in maintaining interpersonal relationships and academic performance (18).

In the economic sphere, victims may develop financial dependence on their aggressors, particularly when the violence includes economic control, and may face medical and legal expenses resulting from treatment or judicial proceedings.

These consequences highlight the importance of recognizing and addressing dating violence (DV) to protect the health and well-being of victims (19).

Various studies have explored factors associated with greater vulnerability to experiencing or perpetrating violence in adolescent dating relationships. Evidence indicates that exposure to family or childhood violence may be associated with a higher likelihood of reproducing harmful dynamics in intimate relationships, not as a direct determinant but as a contextual factor that may influence the construction of relational models (20–22).

Similarly, alcohol or other substance use, as well as adherence to sexist beliefs or traditional gender roles, have been associated with a greater presence of violent behaviors. However, these associations do not imply causality nor define specific groups as predisposed, but rather reflect structural, cultural, and situational conditions that may interact with other individual and social factors (20).

In this regard, gender beliefs that legitimize violence have been linked to both perpetration and victimization, particularly among men, who tend to show greater adherence to these stereotypes (17).

Similarly, low self-esteem and emotional dependence have been described as factors that may hinder the identification of abusive behaviors and, in some cases, affect the ability to end harmful relationships.

It is important to note that students belonging to sexual and gender minorities, particularly those with marginalized intersectional identities, present significantly higher rates of abuse and harassment in dating relationships (23). In the digital sphere, problematic use of social media has given rise to new manifestations of violence, such as constant monitoring, surveillance, or pressure exerted through messages (24).

Conversely, several protective factors may help prevent these situations, including relationship and sexuality education based on equality, family and social support, participation in community or sports activities, and access to prevention and specialized support services. These elements strengthen adolescents’ personal, emotional, and social skills, thereby promoting the development of healthier and more respectful relationships (25).

According to the study by Llano-Suárez et al. (26), conducted with a sample of 1,064 heterosexual female university students in Spain, 20.8% of the participants reported having experienced sexual violence within the context of an intimate relationship. This form of violence often occurs in combination with other types of abuse, such as coercion, humiliation, and physical violence.

In addition, several studies have identified certain factors associated with greater vulnerability to victimization in intimate relationships. Among these, specific relational dynamics and prior relational experiences have been noted, such as affective trajectories characterized by lower stability or by relationships marked by previous inequalities, as well as the presence of violence within the family environment, which may shape relational patterns that hinder the early identification of risk situations. This reality highlights the need to address gender-based violence in intimate relationships from a comprehensive perspective that considers not only individual factors but also the social and cultural attitudes that perpetuate it (27).

In this regard, several studies indicate that attitudes toward gender-based violence may vary according to gender identity and cultural context, and that both men and women may hold beliefs that contribute to its normalization (28).

To the best of our knowledge, no recent quantitative multicenter study has specifically focused on analyzing the degree of perceived gender-based violence during dating among university students in the Health Sciences field. However, the existing literature includes studies addressing both gender identity differences and the prevalence of gender-based violence among university students (21, 29, 30).

In this context, the focus on Health Sciences students is particularly relevant. Health Sciences students constitute a particularly relevant population for the study of dating violence. On the one hand, they are situated within the same developmental stage as other young adults and may therefore be exposed to similar relational dynamics and risks associated with dating violence (31). On the other hand, as future healthcare professionals, they are expected to play a key role in the early identification, prevention, and management of gender-based violence in clinical and community Settings (32, 33).

Evidence suggests that healthcare professionals often encounter victims of gender-based violence in different care contexts; however, a lack of training and awareness may hinder adequate detection and response. In this regard, understanding Health Sciences students’ experiences, perceptions, and potential exposure to dating violence is essential to inform educational strategies aimed at strengthening their competencies in recognising and addressing these situations in their future professional practice (34, 35).

For this reason, the main objective of this study is to determine the prevalence and dynamics of DV among Health Sciences university students in Health Sciences at the Universitat de Barcelona, Universitat Autònoma de Barcelona, Universitat Rovira i Virgili, Campus Docent Sant Joan de Déu, Universitat de Girona, Universitat de Lleida, Universidad del Norte, and Universidad de Cartagena (Colombia).

## 2 Material and methods

### 2.1 Participants

The study population consisted of students enrolled in Health Sciences degree programs from eight universities, six of them in Spain (*n* = 448) and two in Colombia (*n* = 63).

In Spain, five public universities participated: Universitat de Barcelona (*n* = 180), Universitat de Girona (*n* = 55), Universitat Rovira i Virgili (*n* = 160), Universitat Autònoma de Barcelona (*n* = 3), and Universitat de Lleida (*n* = 15). In addition, one publicly funded private institution participated: Campus Docent Sant Joan de Déu (*n* = 35). In Colombia, one public university (Universidad de Cartagena, *n* = 48) and one private university (Universidad del Norte, *n* = 15) were included. All students were enrolled during the academic year from January to December 2024.

The sample size was calculated to ensure sufficient statistical power to detect meaningful differences in the primary outcome variables related to dating violence. Assuming an expected standard deviation of 6 points, a minimum detectable difference of 2 points, a statistical power of 80%, and a significance level of *α* = 0.05, a minimum sample size of 450 participants was estimated. An additional 10% was added to account for potential incomplete data, resulting in a target sample of approximately 505 students. The final analyzed sample consisted of 511 participants.

The inclusion criteria were enrollment in an undergraduate program in Health Sciences and voluntary agreement to participate in the study through signed informed consent. As an exclusion criterion, students who were not enrolled in the continuous assessment modality at the time of data collection were excluded.

The total sample consisted of 511 students selected through intentional or convenience sampling. Of the participants, 17.2% were men and 82.6% were women; this unequal gender distribution reflects the predominance of women in the selected university programs. Participants’ ages ranged from 17 to 50 years (M = 21.50; SD = 5.19).

### 2.2 Instruments

For data collection, the Multidimensional Dating Violence Scale (EMVN), developed by García-Carpintero et al. (36), was used. This scale was designed to detect gender-based violence both perpetrated and experienced in dating relationships among university students and has demonstrated validity and reliability in academic contexts. The EMVN consists of 32 items distributed across three main dimensions: (a) physical and sexual aggression; (b) control behaviors (such as cyberbullying, surveillance, and harassment); and (c) psycho-emotional abuse (such as denigration and domination). Each item is rated using a Likert-type scale, allowing assessment of the frequency or intensity of the behaviors described. The scale considers both the victim's and the perpetrator's perspectives, enabling a comprehensive evaluation of dating violence dynamics. The EMVN has been previously validated in university populations, demonstrating strong psychometric properties, including high internal consistency and construct validity () Garcia carp). In a validation conducted with a university population in Tarapoto (2022), internal consistency coefficients of *α* = 0.990 and *ω* = 0.980 were reported, supporting its use in research with university students.

An *ad hoc* questionnaire was also administered to collect relevant sociodemographic information (age, gender identity, nationality, etc.).

### 2.3 Procedure

The first step in conducting this research was to contact the deans of the faculties at the selected universities to present the objectives of the study and request their collaboration and institutional authorization. Subsequently, communication was established with the coordinators of courses related to biopsychosocial care, who facilitated the implementation of the study within the academic setting. The instructors responsible for the courses shared the access link to the REDCap platform through the virtual campus during academic sessions.

The informed consent form, the *ad hoc* sociodemographic questionnaire, and the Multidimensional Dating Violence Scale (EMVN) were digitized through the REDCap platform. Before accessing the instruments, participants were required to explicitly accept the informed consent. The estimated time to complete the questionnaire was approximately 20 minutes, and data collection was completed after five months.

### 2.4 Data analysis and processing

Data analysis was conducted using descriptive and inferential statistics. First, absolute and relative frequencies were calculated for categorical variables (such as gender, field of study, nationality, and university of origin), as well as measures of central tendency and dispersion (median, interquartile range, minimum, and maximum) for quantitative variables (such as age and scores on the EMVN scale).

Because the distributions did not meet the assumption of normality (Kolmogorov–Smirnov test; *p* < 0.05), the use of nonparametric statistical tests was justified in the subsequent analyses.

To explore possible relationships between participants’ sociodemographic and academic characteristics and their perceived levels of violence, bivariate statistical analyses were conducted. For quantitative variables, Spearman's rank correlation coefficient was used, whereas for categorical variables, the Mann–Whitney U test was applied. In a second phase, multiple linear regression models were conducted to examine the association between the different dimensions of perceived violence. These models were conceived for exploratory and explanatory purposes to quantify the observed associations. To avoid collinearity among the dimensions of dating violence, three independent models were constructed, one for each dimension: (a) physical and sexual aggression; (b) control behaviors (such as cyberbullying, surveillance, and harassment); and (c) psycho-emotional abuse. Each model was adjusted for relevant sociodemographic and academic variables: age, origin, gender identity, university degree program, academic year, faculty, and university of origin. All models assumed a 95% confidence level, and results with p-values below 0.05 were considered statistically significant. All analyses were performed using SPSS statistical software (version 30).

### 2.5 Ethical considerations

This multicenter study was approved by the Research Ethics Committees of all participating institutions. The study was conducted in accordance with the ethical principles of the Declaration of Helsinki, the Council of Europe Convention, and the UNESCO Universal Declaration. All participants provided written informed consent prior to enrollment. Data confidentiality and participant anonymity were ensured in accordance with the General Data Protection Regulation (GDPR) and applicable to local laws. Ethical approvals were obtained from the following committees: Institutional Review Board (IRB00003099), reference number CER062407; CEIm Universitat Rovira i Virgili, reference number CEIPSA-2024-PR-0018; Comité de Ética Universidad del Norte, reference number 2407-7047; Comité de Ética Universidad de Cartagena, reference number 175; Comité de Ética de la Recerca amb Medicaments (CERT 114), Universitat de Lleida; Comité de Ética de la Investigación Biomédica, Universitat de Girona, reference number CEBRU0043-24; and Comité de Ética de la Universitat Autònoma de Barcelona, reference number CEEAH7219.

### 2.6 Reporting method

This manuscript was prepared in accordance with the Strengthening the Reporting of Observational Studies in Epidemiology (STROBE) Statement (File S1) (37).

## 3 Results

### 3.1 Sociodemographic results

The sample consisted of 511 participants. Age ranged from 17 to 30 years, with a mean age of 21.55 years (SD = 5.19). Regarding sex, the majority identified as female (82.6%), whereas 17.2% identified as male and 0.2% preferred not to respond. With respect to reported nationality, most participants were from Spain (82.0%), followed by Colombia (12.5%). The remaining countries each represented less than 1% of the sample, including Algeria, Argentina, Bolivia, Brazil, China, Cuba, Ecuador, France, Georgia, Morocco, Mexico, Paraguay, Peru, Portugal, Romania, Ukraine, and Venezuela.

Regarding the university degree program, most participants were enrolled in Nursing (78.1%), followed by Medicine (10.2%), Biomedicine (6.5%), and Podiatry (5.3%). In terms of academic year, nearly half were in the first year (49.9%), followed by the second year (19.6%) and the third year (16.2%). Smaller percentages were observed in the fourth year (6.3%), sixth year (7.6%), and fifth year (0.4%). Finally, with respect to the university of origin, the largest proportion corresponded to Universitat de Barcelona (35.2%) and Universitat Rovira i Virgili (31.3%). Students also participated from Universidad de Cartagena (9.4%), Universitat de Girona (10.8%), and Fundació Sant Joan de Déu Campus Docent (6.8%). The remaining universities represented percentages below 3%, including Universidad del Norte, Universitat de Lleida, and Universitat Autònoma de Barcelona.

### 3.2 EMVN scale results

Regarding the EMVN, the mean score on the victimization subscale was M = 49.00 (IQR = 8), with values ranging from 18 to 60. The perpetration subscale showed a mean score of M = 23.00 (IQR = 5), with a range between 18 and 51. Concerning the specific dimensions of the victimization subscale, the highest scores were observed in control and surveillance (M = 7.00; IQR = 2), sexual violence (M = 5.00; IQR = 0), and cyberbullying (M = 5.00; IQR = 3), whereas the lowest score corresponded to physical violence (M = 2.00; IQR = 0). The psycho-emotional dimension showed lower values than cyberbullying (M = 4.00; IQR = 2). In the perpetration subscale, the highest scores were recorded in control and surveillance (M = 7.00; IQR = 2), sexual violence (M = 5.00; IQR = 0), and cyberbullying (M = 4.73; IQR = 2), whereas the lowest score corresponded to physical violence (M = 2.00; IQR = 0). The psycho-emotional dimension showed lower values than both sexual violence and cyberbullying (M = 4.00; IQR = 2). Table 1 presents the medians and interquartile ranges observed in the EMVN by subscale and dimension.

**Table 1:** Descriptive statistics of the EMVN scale (victimization and perpetration).

Dimension/subscale	N	Min	Max	Cutoff point	Median	IQR
Total victimization	511	18	60	—	49.00	8
Total perpetration	511	18	51	—	23.00	5
Victimization - Cyberbullying	511	3	12	8	5.00	3
Victimization - Control/surveillance	511	5	17	10	7.00	2
Victimization – Psycho-emotional abuse	511	3	12	6	4.00	2
Victimization – Physical violence	511	2	6	3	2.00	0
Victimization – Sexual violence	511	5	12	8	5.00	0
Perpetration - Cyberbullying	511	3	11	8	5.00	2
Perpetration - Control/surveillance	511	5	15	10	7.00	2
Perpetration - Psycho-emotional abuse	511	3	12	6	4.00	2
Perpetration – Physical violence	511	2	6	3	2.00	0
Perpetration – Sexual violence	511	5	12	8	5.00	0

EMVN = Dating Violence Scale (*Escala de Maltrato en el Noviazgo*). Values correspond to the medians and interquartile ranges (IQR) for each subscale and dimension derived from the descriptive analysis conducted in SPSS.

To contextualize these results and ensure the robustness of the measures used, the reliability of both subscales was evaluated. The internal consistency analysis showed adequate Cronbach's alpha values for both subscales: victimization (*α* = 0.805) and perpetration (*α* = 0.707), indicating good reliability of the instrument in the study sample.

### 3.3 Distribution of scores by item

In the victimization subscale, the items with the highest mean scores were “Giving unsolicited gifts or favors” (M = 2.00; IQR = 2) and “Insisting on sending messages via WhatsApp or other social networks” (M = 2.00; IQR = 1), corresponding to the dimensions of control and surveillance and cyberbullying, respectively. In contrast, the items with the lowest scores were concentrated in the physical violence and sexual violence dimensions, particularly “Severe physical assault” (M = 1.00; IQR = 0) and “Taking advantage of intoxication” (M = 1.00; IQR = 0), indicating a low prevalence of these behaviors.

In the perpetration subscale, a similar pattern was observed: the items with the highest mean scores were “Giving unsolicited gifts or favors” (M = 2.00; IQR = 2) and “Insisting on sending messages via WhatsApp or other social networks” (M = 1.00; IQR = 1), whereas the least frequent behaviors corresponded to the sexual dimension, such as “Taking advantage of intoxication” (M = 1.00; IQR = 0) and “Engaging in sexual touching without consent” (M = 1.00; IQR = 0).

In both subscales, a high floor effect was observed in most items, particularly those related to physical violence (above 95%), indicating that the majority of participants reported neither experiencing nor perpetrating these behaviors. The ceiling effect was virtually nonex suggesting no saturation in the highest response categories (Table 2).

**Table 2:** Descriptive statistics of the eMVN by item and dimension. .

Dimension	Item	Victimization	Perpetration
		M, IQR, Min–Max, %Floor, %Ceiling	M, IQR, Min–Max, %Floor, %Ceiling
Cyber bullying	1. Insistently sending WhatsApp's or other type of message on social networks	2.00, 1, 1–4, 42.1%, 2.5%	1.00, 1, 1–4, 98.8%, 0%
	2. Spying on the other person's activity on the networks	1.00, 1, 1–4, 54.4%, 1.6%	1.00, 1, 1–4, 97.8%, 0%
Control & surveillance	3. Monitoring the time of the other person's last connection	1.00, 1, 1–4, 61.4%, 1.4%	1.00, 1, 1–4, 62.4%, 1.0%
	4. Giving gifts or favors not asked for	2.00, 2, 1–4, 28.4%, 7.2%	2.00, 2, 1–4, 97.1%, 0.8%
	5. Going on purpose past where the other person usually is	1.00, 1, 1–4, 71.8%, 0.2%	1.00, 1.00, 1–3, 72.6%, 0%
	6. Asking where he/she is every minute of the day	1.00, 0, 1–4, 76.7%, 0.4%	1.00, 0, 1–4, 85.3%, 0.2%
	7. Trying to make the other person feel guilty	1.00, 1, 1–4, 66.5%, 0.6%	1.00, 0, 1–4, 82.0%, 0.4%
	8. Checking through friends or family	1.00, 0, 1–4, 82.8%, 0.2%	1.00, 0, 1–3, 88.6%, 0%
Psycho-emotional	9. Bringing up something from the past to hurt	1.00, 1, 1–4, 58.5%, 1.4%	1.00, 1, 1–4, 68.5%, 0.4%
	10. Blaming him/her for things that don't go well	1.00, 1, 1–4, 63.6%, 0.6%	1.00, 1, 1–4, 71.8%, 0.4%
	11. Avoiding or refusing to talk when angry	1.00, 1, 1–4, 54.6%, 1.6%	1.00, 1, 1–4, 60.7%, 0.8%
Physical	12. Physically harming someone they know	1.00, 0, 1–3, 96.5%, 0%	1.00, 0, 1–3, 97.8%, 0%
	13. Serious physical attack	1.00, 0, 1–3, 97.7%, 0%	1.00, 0, 1–3, 97.8%, 0%
Sexual	14. Not asking for consent to sexual intercourse	1.00, 0, 1–4, 93.5%, 0.8%	1.00, 0, 1–4, 97.1%, 0.8%
	15. Taking advantage of intoxication	1.00, 0, 1–2, 97.3%, 0%	1.00, 0, 1–3, 99.0%, 0%
	16. Asking for unwanted sexual practices	1.00, 0, 1–3, 93.3%, 0%	1.00, 0, 1–2, 98.8%, 0%
	17. Pressuring someone to have sex without a condom	1.00, 0, 1–4, 86.9%, 0.2%	1.00, 0, 1–3, 97.5%, 0%
	18. Engaging in sexual touching without permission	1.00, 0, 1–3, 98.8%, 0%	1.00, 0, 1–2, 98.8%, 0%

In the multiple linear regression analysis between the different sociodemographic and academic variables and the perception of violence, no significant differences or associations were found, except for gender identity and context, where relevant differences were identified. In the perpetration subscale, men reported higher levels of physical violence [mean difference = 0.130; 95% CI (0.053, 0.207); *p* < .001] and sexual violence [mean difference = 0.190; 95% CI (0.055, 0.324); *p* = .006] compared with women. In the cyberbullying perpetration subscale, Spain showed higher values than Colombia [difference = 0.583; 95% CI (0.196, 0.970); *p* = .003]. At the local level, students from Universitat de Lleida reported more physical violence behaviors than those from other Catalan universities (*p* < .001), and cyberbullying was more prevalent in Catalan contexts compared with Colombia (*p* < .01) (Table 3).

**Table 3:** Analysis of multiple regression linear (dimensions*)*.

Outcome	Mean difference		
	(M–F)	95% CI	*p*-value
Victimisation – Cyberbullying	0.346	[-0.058, 0.751]	0.093
Victimisation – Control/surveillance	0.197	[-0.250, 0.644]	0.387
Victimisation – Psychoemotional	0.350	[-0.015, 0.715]	0.060
Victimisation – Physical	0.130	[0.053, 0.207]	**<0.001**
Victimisation – Sexual	-0.053	[-0.284, 0.178]	0.652
Perpetration – Cyberbullying	0.050	[-0.292, 0.392]	0.774
Perpetration – Control/surveillance	-0.194	[-0.572, 0.184]	0.313
Perpetration – Psychoemotional	0.092	[-0.215, 0.399]	0.556
Perpetration – Physical	0.023	[-0.046, 0.092]	0.510
Perpetration – Sexual	0.190	[0.055, 0.324]	**0.006**

Values represent mean differences between male and female participants (M–F). Confidence intervals (CI) and *p-values* are reported.

Significant results (*p* < .05) are shown in bold. Analyses are based on estimated marginal means with LSD adjustment.

## 4 Discussion

The aim of this study was to determine the degree of dating violence (DV) among Health Sciences university students from Spanish and Colombian universities. This descriptive, cross-sectional, multicenter study provides a detailed overview of the prevalence and characteristics of DV among students from different regions and sociocultural contexts. The results revealed a notable presence of violent behaviors, particularly in the victimization subscale, whose scores were higher than those observed for perpetration. The mean victimization scores showed elevated levels, with a particularly high incidence in the dimensions of control and surveillance and sexual violence, suggesting that these forms of violence, often more subtle or socially normalized, are perceived more frequently by participants. However, it is important to note that higher scores in control and surveillance behaviours do not necessarily indicate that participants explicitly recognise these behaviours as violence. Rather, these findings may reflect both their frequency and a potential lack of awareness or identification of such behaviours as abusive, which has important clinical implications.

Similarly, the perpetration subscale showed a comparable pattern, with higher values in the same dimensions and lower scores in physical violence. This parallelism between victimization and perpetration reinforces the idea of a bidirectional relational dynamic in which psychological and sexual forms of violence predominate physical manifestations.

The findings of our study converge with previous evidence identifying dating violence as a highly prevalent and persistent phenomenon in higher education institutions (38, 39). Various international studies highlight that, particularly among Health Sciences students, this issue has become a widespread occurrence that often remains under detected. In Spain, for example, a study among nursing students found that 53% had experienced and/or perpetrated DV, and around 20% reported violence through social networks or cyberviolence (39). Similarly, among Spanish nursing and other Health Sciences students, 61.9% had experienced at least one abusive behavior, with psychological manifestations predominating (40). Likewise, in a comparable context such as Portugal, 53.7% of students reported having experienced an episode of DV (28).

In geographically diverse contexts with different sociocultural frameworks, a pattern comparable to that observed in the present study has been reported. Among nursing and midwifery students in Turkey, a high prevalence of dating violence was identified, particularly psychological and verbal forms, although a significant proportion of students did not recognize these behaviors as violence, suggesting difficulties in identifying and normalizing such dynamics (41). In Nepal, 78% of public health students with experience in romantic relationships reported lifetime DV (42). In Mexico, medical students reported substantial levels of violence and cyberaggression; moreover, the acceptance of emotional violence was associated with an increased risk of experiencing physical violence (43).

When focusing on the Colombian context, the findings of the present study are broadly consistent with previous research conducted in this setting. Studies such as Rey-Anacona (12), Pérez-Ruiz et al. (13), and Rey-Anacona et al. (14) have reported high prevalence rates of dating violence, particularly in psychological and controlling behaviours. These studies also highlight challenges in the recognition and normalization of certain forms of violence, which is consistent with the patterns observed in the Colombian subsample of the present study.

Although some studies report apparently lower prevalence rates, for example, 3.1% of DV in a Turkish Health Sciences sample, the authors themselves suggest that these figures may be due to difficulties in recognizing, defining, and reporting violent behaviors rather than to a genuinely lower presence of the phenomenon (44).

In this regard, the specialized literature consistently indicates that psychological violence constitutes a central and pervasive component of dating violence, to the extent that it is often present in most cases, either as the primary form or as a dimension accompanying other manifestations of violence (21). Its subtle, progressive nature and, in many contexts, its social normalization make it a key factor in the development and maintenance of harmful relational dynamics.

In this same line, the results of this study confirm that psychological violence is widely present among the participating students. However, our findings refine this general pattern: the dimensions with the highest scores were control and surveillance, which form part of psychological violence, and secondly sexual violence, suggesting that controlling behaviors and sexually coercive dynamics emerge as the most predominant in this sample. This pattern is consistent with previous literature, which consistently identifies psychological violence and controlling behaviors as the most prevalent forms of abuse across different geographic contexts (21). In Portugal, for example, more than 50% of university students reported having experienced this type of violence, manifested through humiliation, devaluation, emotional detachment, and controlling behaviors (28, 30). Similarly, in Ecuador this type of aggression is also the most frequently reported, affecting 56.9% of women according to national data cited in the study (45). Currently, these dynamics are increasingly mediated through technology, including constant monitoring, threats, and harassment through social networks and messaging applications (10). Several studies indicate that these experiences are highly prevalent and closely linked both to episodes of offline violence and to a significant deterioration in mental health. In Spain, for example, more than half of nursing students reported having been involved in situations of DV, and approximately 20–22% reported having perpetrated or experienced violence through social networks (39). Similarly, among first-year medical students in Mexico, the coexistence of face-to-face violence and cyberaggression was observed; moreover, men reported cyber-victimization more frequently, whereas women and sexual minorities presented a higher risk of gender-based violence and offline DV (15).

Regarding gender identity differences in dating violence, the evidence, consistent with our findings, indicates differentiated patterns depending on the type of aggression. Several studies consistently report that women more frequently report victimization due to sexual violence, including coercion and unwanted sexual contact (38). Likewise, men report higher levels of perpetration, whereas women report greater victimization (46). Although overall rates of aggression may appear similar across genders, the literature highlights that men tend to cause more severe injuries and do so more frequently than women (29). With respect to psychological violence, our findings indicate that no significant differences were observed between gender identities, a pattern that aligns with previous studies. For example, a university sample reported very high levels of bidirectional psychological perpetration, with similar rates among women and men and no overall gender identity differences (47). In addition, psychological “micro-abuses”, such as belittlement, role imposition, or invasion of personal space, are also described as frequent behaviors in both directions, with students of both gender identities reporting having both perpetrated and experienced them (48).

Similarly, the literature shows that these patterns are not uniform across countries. In several Latin American contexts, for example, higher rates of physical and psychological violence perpetration have been documented compared with European or Asian regions (5). When focusing on rural settings, our findings are consistent with evidence indicating a greater likelihood of experiencing violence compared with urban areas (49). Likewise, with respect to cyberbullying, an increasingly prevalent phenomenon, our findings show a differential trend compared with that described in other studies. In comparisons between Spain and several Latin American countries, overall involvement rates in cyberbullying have been found to be higher in Ecuador, Colombia, and Uruguay than in Spain (50). In addition, another European study identified Spain as among the countries with lower prevalence rates of cyberbullying, in contrast to others such as Italy or Greece (51). Observed differences between Spain and Colombia should be interpreted not only in epidemiological terms, but also in relation to legislative and cultural factors. The Spanish legislative framework (Organic Law 1/2004 and Law 17/2020) may contribute to a greater identification of subtle and psychological forms of abuse, including digital controlling behaviours. In contrast, the Colombian legal framework (Law 1257 of 2008), together with its sociocultural context—characterised by greater structural inequalities—may explain the coexistence of higher levels of explicit forms of violence alongside lower sensitivity to certain abusive behaviours due to their normalisation (52). Furthermore, previous research has shown that Spanish adolescents tend to perceive dating violence more accurately than their Colombian counterparts, highlighting the role of cultural context in the identification and reporting of abusive situations (53).

These results also align with perspectives from feminist scholarship and gender studies, which emphasize that gender-based violence and violence within intimate relationships are structural phenomena strongly shaped by specific sociocultural contexts. In this regard, future research should further examine how specific contextual factors, such as rural versus urban settings, may influence not only the prevalence but also the perception, recognition, and normalization of these behaviours.

### 4.1 Limitations

The results of this study should be interpreted considering several limitations. First, the cross-sectional design prevents the analysis of temporal changes in the behaviors studied and therefore does not allow assessment of how this may have influenced the findings. In addition, the use of a convenience sample limits the representativeness of the university student population, as the sample included a substantially higher proportion of women than men, requiring cautious interpretation of the results. It should also be noted that participation across areas of knowledge was uneven, which limited comparability between disciplines. Finally, the use of a self-administered questionnaire may have introduced biases related to systematic response patterns. Nevertheless, these limitations will be considered in future research, and replication of these studies in other contexts would be important to obtain a clearer understanding of dating violence. Likewise, future lines of research should consider sexual orientation as a relevant variable for examining associations with the instruments analyzed.

The results of this study should be interpreted considering several limitations. First, the cross-sectional design prevents the analysis of temporal changes in the behaviors studied and therefore does not allow assessment of causal relationships or the directionality of the observed associations. In addition, the use of a convenience sample limits the representativeness of the university student population, as the sample included a substantially higher proportion of women than men, requiring cautious interpretation of the results. Furthermore, although participants were drawn from Health Sciences programmes, the sample was predominantly composed of nursing students and women, which may limit the generalisability of the findings to other academic disciplines and to more gender-diverse populations. It should also be noted that participation across areas of knowledge was uneven, which limited comparability between disciplines. Another limitation relates to the measurement instrument used. Although the EMVN has demonstrated adequate psychometric properties in previous studies, it has not been specifically validated in the Colombian context. This may affect the cross-cultural comparability and interpretation of the findings. Finally, the use of a self-administered questionnaire may have introduced biases related to systematic response patterns. Nevertheless, these limitations will be considered in future research, and replication of these studies in other contexts would be important to obtain a clearer understanding of dating violence. Likewise, future lines of research should consider sexual orientation as a relevant variable for examining associations with the instruments analyzed.

## 5 Conclusions

In summary, it is essential to emphasize the importance of continuing research within the university context, as these institutions must consolidate their role as spaces that guarantee egalitarian relationships and promote health. The results obtained show a significant presence of dating violence (DV), particularly in the dimensions of surveillance and control, as well as sexual violence. These findings may serve as a basis for the development of equality strategies and health promotion plans within universities. In this context, nursing could play a particularly relevant role due to its training and expertise in relation to gender-based violence and health.

The implementation of these initiatives could contribute significantly to improving the quality of life of the university community and, in the long term, generate a positive impact on society. However, it is essential to advance through new lines of research, particularly qualitative studies, that allow for deeper exploration of individual processes and lived experiences, as well as guide the design of specific interventions across different Health Sciences degree programs that can subsequently be evaluated. In this regard, it is essential to conduct research from a feminist perspective capable of making structural inequalities visible and situating violence within the framework of gender power relations, thereby ensuring a more comprehensive and transformative approach to the phenomenon.

### 5.1 Implications for practice

This study provides relevant evidence regarding the knowledge and magnitude of gender-based violence in dating relationships among Health Sciences students. This group plays a strategic role, as within their professional disciplines they are not only responsible for raising awareness in society but must also be specifically trained in the identification and care of victims. In this sense, the objective of academic training is not only to sensitize students but also to equip them with the necessary tools to intervene professionally, while at the same time making them aware of their own relationships and of the potential abuse they themselves may be experiencing. In this regard, specific curricular interventions could include simulation-based training for the identification and management of dating violence, reflective learning activities aimed at challenging beliefs that may normalize abusive behaviours, and the integration of dedicated modules on gender-based violence within Health Sciences curricula to strengthen students’ clinical and interpersonal competencies.

The multicenter approach of the study, which includes the participation of Spain and Colombia, makes it possible to identify significant cultural differences that can guide the design of more contextualized interventions sensitive to the sociocultural particularities of each setting. Likewise, the study contributes to making visible a problem that is often normalized in the university context. This normalization is sustained by the naturalization and integration of everyday sexist behaviors which, when perceived as ordinary, prevent victims and their surroundings from identifying certain behaviors as abuse.

Finally, the data obtained may inform the updating of institutional protocols for detection and referral, as well as contribute to the development of public policies aimed at improving institutional and community responses to gender-based violence among young people.

## Data Availability

The raw data supporting the conclusions of this article will be made available by the authors, without undue reservation.
